# Antibiotic exposure for suspected early-onset sepsis in late-preterm and full-term newborns in Quebec: a 11-year single-center cohort study

**DOI:** 10.3389/fped.2026.1922279

**Published:** 2026-09-09

**Authors:** Varvara Dimopoulou, Brigitte Martin, Christian Lachance, Julie Blackburn, Sophie Tremblay, on behalf of the Q-NEAS study group

**Affiliations:** 1 Clinic of Neonatology, Department Mother-Woman-Child, Lausanne University Hospital and University of Lausanne, Lausanne, Switzerland; 2 Department of Pharmacy, Sainte-Justine University Hospital, University of Montreal, Montreal, QC, Canada; 3 Azrieli Research Center, Sainte-Justine University Hospital, Montreal, QC, Canada; 4 Department of Pediatrics, Division of Neonatology, Sainte-Justine University Hospital, University of Montreal, Montreal, QC, Canada; 5 Department of Pediatrics, Division of Infectious Diseases, Sainte-Justine University Hospital, University of Montreal, Montreal, QC, Canada

**Keywords:** antibiotics, antimicrobial stewardship, early-onset sepsis, late-preterm and term neonates, newborn

## Abstract

**Background:**

Antibiotic exposure during the first postnatal week is common among late-preterm and term newborns and represents an important target for neonatal antimicrobial stewardship. We aimed to quantify antibiotic exposure, assess temporal trends, and determine the incidence and mortality of culture-proven early-onset sepsis (CP-EOS) in a Canadian tertiary care center.

**Methods:**

This retrospective cohort study included all inborn neonates ≥34 weeks' gestation born at a Canadian tertiary care center between 2013 and 2023. Data were extracted from electronic medical records and institutional microbiology and pharmacy databases. Primary outcomes were antibiotic exposure during the first postnatal week and treatment duration. Secondary outcomes included the incidence and mortality of CP-EOS. Antibiotic exposure indicators were calculated using standardized international benchmarking metrics.

**Results:**

Among 34,668 late-preterm and term newborns born during the study period, 1,824 (5.3%; 95% CI, 4.5%–6.0%) received intravenous antibiotics during the first postnatal week for suspected early-onset sepsis (EOS). The median (IQR) antibiotic duration was 3 (3–4) days overall, 9 (5–14) days among neonates with CP-EOS, and 3 (3–5) days among those without proven EOS. Overall antibiotic exposure corresponded to 238 (IQR, 218–259) antibiotic days per 1,000 livebirths. CP-EOS incidence was 0.55 per 1,000 livebirths (95% CI, 0.24–0.83). Overall, for each confirmed EOS case, 96 newborns received antibiotics, and 433 antibiotic days were administered.

**Conclusions:**

Antibiotic exposure substantially exceeded the burden of CP-EOS. Standardized benchmarking indicators may help guide neonatal antimicrobial stewardship efforts.

## 1 Introduction

Neonatal early-onset sepsis (EOS) is a severe condition associated with substantial morbidity and mortality (1). In case of culture-proven EOS (CP-EOS) initiation of antibiotic therapy is undoubtfully lifesaving (2). Among late-preterm and term newborns, the incidence of culture-proven EOS (CP-EOS) remains low, estimated at approximately 0.5 cases per 1,000 livebirths in high-income countries (3–8). Despite this relatively low incidence, empirical antibiotic exposure during the first postnatal week remains common (9–11).

This discrepancy largely reflects the diagnostic uncertainty surrounding neonatal EOS. Clinical manifestations are nonspecific, laboratory biomarkers lack optimal sensitivity and specificity, and treatment decisions frequently need to be made before microbiological confirmation becomes available (12–15). Consequently, many clinically stable newborns receive empirical antibiotic therapy despite ultimately having no confirmed infection (9–11, 15).

The increasing recognition of adverse consequences associated with unnecessary neonatal antibiotic exposure has intensified interest in antimicrobial stewardship strategies. Early-life antibiotic exposure alters the developing microbiome and has been associated with increased risks of antimicrobial resistance, prolonged hospitalization, impaired breastfeeding, fungal infections and potentially adverse long-term health outcomes (16–24). Therefore, safely reducing unnecessary antibiotic exposure during the neonatal period has become a major priority.

Recent large international studies have proposed standardized benchmarking indicators to quantify neonatal antibiotic exposure relative to EOS burden (11, 25). These indicators include the proportion of newborns exposed to antibiotics, treatment duration, antibiotic days per 1,000 livebirths, incidence of EOS, mortality, and the number of treated newborns and antibiotic days per EOS case. Such metrics facilitate meaningful benchmarking across centers and support targeted quality improvement initiatives (26).

In this retrospective cohort study, we applied these standardized benchmarking indicators to quantify antibiotic exposure and evaluate temporal trends in relation to the incidence and outcomes of CP-EOS among late-preterm and term newborns born at a Canadian tertiary care center over an 11-year period.

## 2 Materials and methods

### 2.1 Study design and population

The **Q**uebec **N**eonatal **E**arly-onset sepsis and **A**ntibiotic exposure **S**tudy (Q-NEAS) is a retrospective observational cohort study, conducted at Sainte-Justine University Hospital (CHUSJ), a tertiary care center in Montreal, Quebec, Canada. CHUSJ is Canada's largest mother–child academic hospital and a quaternary perinatal referral center. It cares for approximately 4,000–5,000 livebirths annually, including a high proportion of high-risk pregnancies, and its Level III/IV NICU admits extremely preterm infants, neonates requiring complex medical or surgical care, and referrals transferred from hospitals across Quebec.

All live-born neonates born at ≥34 weeks' gestation between January 1, 2013, and December 31, 2023, were eligible for inclusion. Outborn infants were excluded. The study period was selected to allow comparison and benchmarking with previously published international cohorts and to ensure accrual of a sufficiently large population to generate stable estimates of antibiotic exposure and EOS incidence (11).

The study was approved by the institutional Research Ethics Committee of CHUSJ (CER 2025-8474). The requirement for written informed consent was waived owing to the retrospective and observational nature of the study. The study was conducted in accordance with the Strengthening the Reporting of Observational Studies in Epidemiology for Newborn Infection (STROBE-NI) guidelines (27).

### 2.2 Data collection

Data were extracted from institutional electronic medical records and microbiology and pharmacy databases. Demographic variables included sex, gestational age, and birth weight. Additional variables included duration of antibiotic therapy, microbiological culture results, mortality, and annual birth rates. Urine cultures were not included in the definition of CP-EOS, as they are not routinely recommended as part of the diagnostic evaluation for early-onset neonatal infection and were not systematically collected in the AENEAS dataset (2).

### 2.3 Definitions

CP-EOS was defined as growth of a pathogenic organism in blood and/or cerebrospinal fluid (CSF) cultures obtained within the first postnatal week of life (day 0 to day 6) in association with clinical signs compatible with sepsis and systemic antibiotic treatment for at least five days, unless treatment was interrupted earlier because of death (11, 28, 29). In our institution, we use a 7-day definition of EOS, consistent with the more recent Canadian Paediatric Society guideline. This definition was consistently applied throughout the study period (2013–2023) (29). Organisms usually considered as contaminants, including coagulase-negative staphylococci, diphtheroids and Micrococcus species were excluded. Cases with positive cultures treated for fewer than five days were also excluded. Neonates receiving antibiotic therapy without culture-confirmed infection were classified as having no proven EOS. Antibiotic exposure was defined as administration of intravenous antibiotics initiated during the first postnatal week (day 0 to day 6) for any indication, expressed in antibiotic days per 1,000 livebirths. All-cause neonatal mortality was defined as death before hospital discharge or death within the first 28 postnatal days for infants hospitalized beyond 28 days. EOS-associated mortality was defined as death occurring within 28 days following a positive blood or CSF culture.

During the study period, decisions regarding initiation of empiric antibiotics were based on assessment of maternal risk factors and neonatal clinical condition. Since 2017, management has followed the recommendations of the Canadian Paediatric Society statement on the management of infants at increased risk for sepsis (29). Prior to 2017, the institutional approach was consistent with these principles, with greater reliance on clinical judgment. During the study period, the Kaiser Permanente Neonatal Early-Onset Sepsis Calculator was not systematically used at our institution. Universal maternal Group B Streptococcus (GBS) screening was implemented throughout the entire study period and no major changes to GBS screening practices occurred during the study timeframe.

### 2.4 Outcomes

The primary outcomes were the proportion of neonates exposed to intravenous antibiotics during the first postnatal week, duration of therapy and antibiotic exposure expressed as antibiotic days per 1,000 livebirths. Secondary outcomes included incidence of CP-EOS, all-cause mortality, EOS-associated mortality, the number of treated neonates per EOS case, and the number of antibiotic days administered per EOS case. Temporal trends in antibiotic exposure over the study period were also assessed.

### 2.5 Statistical analysis

The proportion of newborns treated with antibiotics was calculated by dividing the number of newborns receiving at least 1 dose of intravenous antibiotics within the first postnatal week by the number of livebirths. Duration of antibiotic therapy was defined as calendar days with at least 1 dose of antibiotics. Antibiotic exposure was calculated as the sum of antibiotic days of each treated newborn divided by the number of livebirths and was reported as the number of antibiotic days per 1,000 livebirths. Incidence of CP-EOS was defined as the rate in all live-born neonates. Mortality was defined as all events of death for all live-born neonates independent of antibiotic therapy and CP-EOS (overall mortality), and in subgroups (CP-EOS and no EOS).

Continuous variables are presented as medians with interquartile ranges (IQRs), and categorical variables as frequencies with percentages and corresponding 95% confidence intervals (CIs). Temporal trends across calendar years were assessed using Jonckheere–Terpstra tests for continuous variables and Mantel–Haenszel trend tests for categorical variables. Statistical significance was defined as a two-sided *P* value <0.05. All statistical analyses were performed using R software version 4.2.2 (R Foundation for Statistical Computing, Vienna, Austria).

## 3 Results

### 3.1 Study population

A total of 34,668 late-preterm and term newborns were born at CHUSJ between January 1, 2013, and December 31, 2023. Among these neonates, 1,824 (5.3%; 95% CI, 4.5%–6.0%) received intravenous antibiotics during the first postnatal week. Treated newborns included 1,086 boys (60%), with a median (IQR) gestational age of 38 (36–39) weeks and median (IQR) birth weight of 3,140 (2,630–3,560) g. Overall, 19 neonates were diagnosed with CP-EOS, whereas 1,805 received antibiotic therapy without proven EOS (Table 1).

**Table 1:** Demographic characteristics of infants treated with intravenous antibiotics during the first postnatal week.

Characteristics	All (*n* = 1,824)	CP-EOS (*n* = 19)	No proven EOS (*n* = 1,805)
Gestational age, median (IQR), week	38 (36–39)	39 (38–40)	38 (36–39)
Birthweight, median (IQR), g	3,140 (2,630–3,560)	3,420 (3,098–3,876)	3,140 (2,625–3,560)
Male sex, *n* (%)	1,086 (60)	12 (63)	1,074 (60)
Mortality, *n* (%)^a^	31 (1.7)	1 (5.3)	30 (1.7)

CP-EOS, culture-proven early-onset sepsis.

CP-EOS was defined as sepsis within 6 days of birth (day 0–6).

Categorical and continuous variables are displayed as frequencies (percentages), and as median (IQR), respectively.

a Among the 31 deceased patients, 1 (3%) was due to CP-EOS, 19 (61%) deaths were attributable to severe congenital malformations, three (10%) to perinatal asphyxia, and eight (26%) to other causes.

### 3.2 Burden of antibiotic exposure

**Figure 1:**
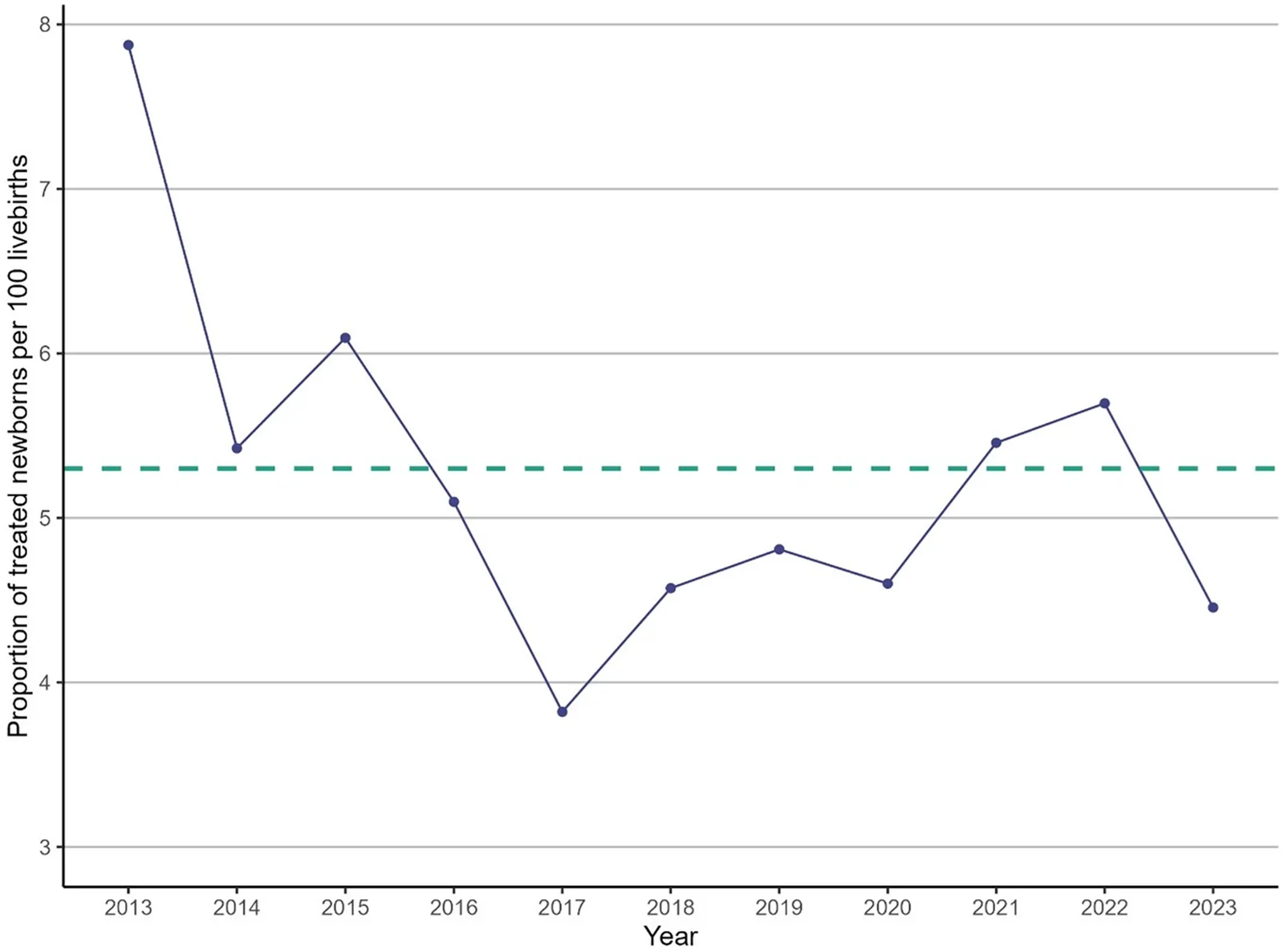
Temporal trends in the proportion of newborns treated with intravenous antibiotics during the first postnatal week. Annual proportion of liveborn infants receiving intravenous antibiotics during the first postnatal week over the study period. Each point represents one calendar year, and the dashed horizontal line indicates the median proportion across the study period (5.3 treated newborns per 100 livebirths).

**Figure 2:**
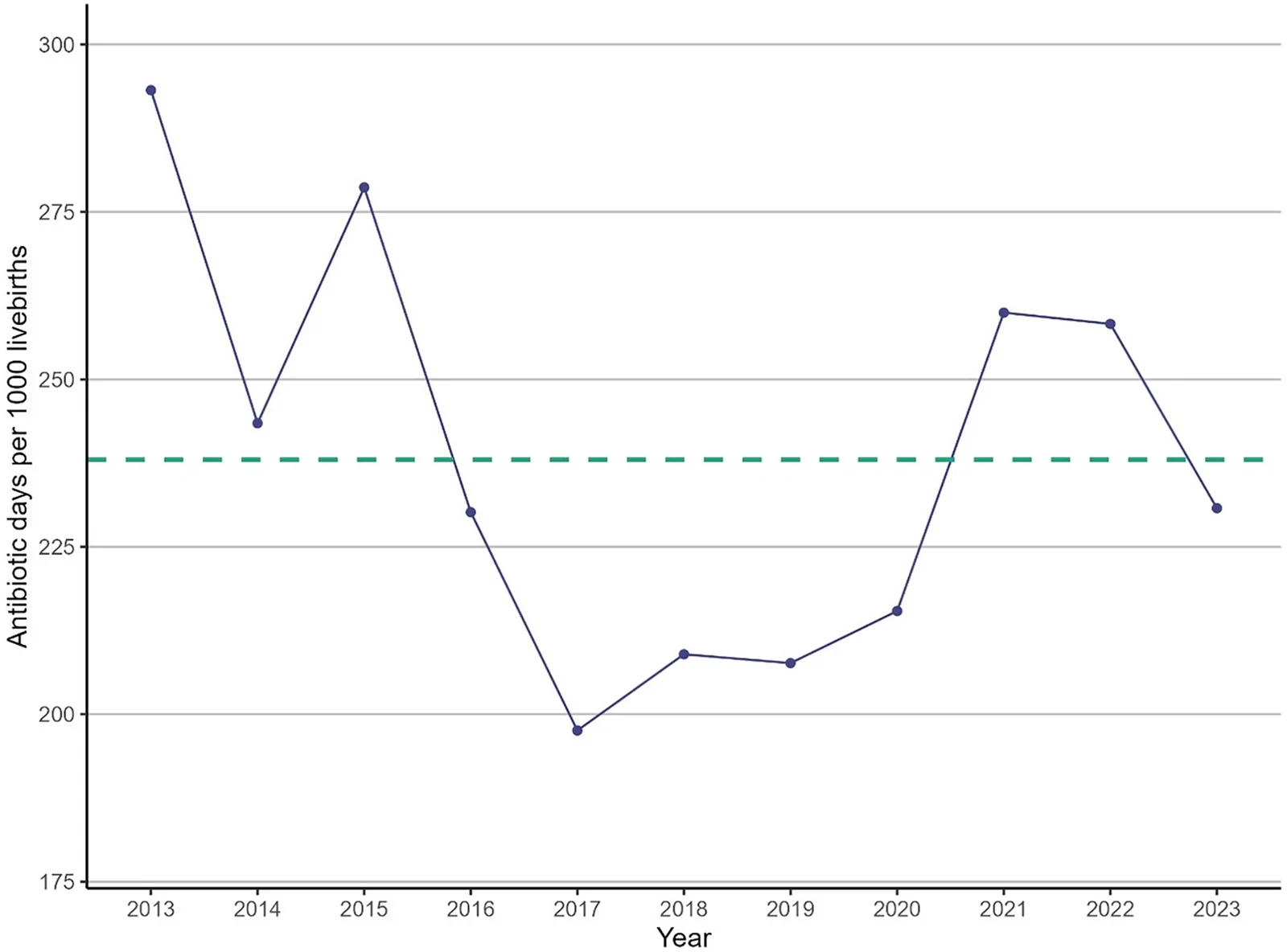
Temporal trends in antibiotic exposure among newborns, expressed as antibiotic days per 1,000 livebirths, from 2013 to 2023. Annual antibiotic exposure among newborns during the study period, expressed as total antibiotic days per 1,000 livebirths from 2013 to 2023. Each point represents one calendar year, and the connecting line illustrates the temporal trend in antibiotic utilization over time.

The median (IQR) duration of antibiotic therapy was 3 (3–4) days for all treated newborns, 9 (5–14) days for those with CP-EOS, and 3 (3–5) days for those without proven EOS. The median (IQR) antibiotic exposure was 238 (218–259) antibiotic days per 1,000 livebirths. Over the study period, the proportion of treated newborns showed an overall downward trend, decreasing by 44%, from 8.0% in 2013 to 4.5% in 2023 (Figure 1), although this decrease was not linear, with year-to-year variability. Similarly, antibiotic exposure decreased by 21%, from 293 to 231 antibiotic days per 1,000 livebirths (Figure 2).

### 3.3 Burden of the disease

CP-EOS was diagnosed in 19 neonates, corresponding to an incidence of 0.55 cases per 1,000 livebirths (95% CI, 0.24–0.83). The incidence of CP-EOS decreased by approximately 72% during the study period, from 1.27 cases per 1,000 livebirths in 2013 to 0.35 cases per 1,000 livebirths in 2023 (Table 2).

**Table 2:** Main outcomes among newborns treated with antibiotics during the first week of life, 2013 to 2023.

Outcomes	All years *n* = 34,668	2013 *n* = 3,162	2014 *n* = 3,282	2015 *n* = 3,298	2016 *n* = 3,315	2017 *n* = 3,219	2018 *n* = 3,302	2019 *n* = 3,140	2020 *n* = 3,217	2021 *n* = 3,262	2022 *n* = 2,598	2023 *n* = 2,873	*P* ^a^
Treated newborns, *n* (%)	1,824 (5.3)	249 (7.9)	178 (5.4)	201 (6.1)	169 (5.1)	123 (3.8)	151 (4.6)	151 (4.8)	148 (4.6)	178 (5.5)	148 (5.7)	128 (4.5)	**0.018**
Duration of antibiotic treatment, median (IQR), d													
All treated	3 (3–4)	3 (3–4)	3 (3–4)	3 (3–4)	3 (2–5)	3 (2–6)	3 (2–4)	3 (2–4)	3 (3–5)	3 (3–5)	3 (3–5)	3 (3–6)	NA
CP-EOS	9 (5–14)	10 (9–12)	9 (8–10)	12 (12–12)	7 (6–11)	-	9 (6–12)	3 (2–3)	-	13 (9–14)	-	14 (14–14)	0.533
No-Sepsis	3 (3–4)	3 (3–4)	3 (3–4)	3 (3–4)	3 (2–5)	3 (2–6)	3 (2–4)	3 (2–4)	3 (3–5)	3 (3–5)	3 (3–5)	3 (3–6)	NA
Antibiotic days, No.	8,261	927	799	919	763	636	690	652	693	848	671	663	NA
Antibiotic days/1,000 livebirths, No.	238	293	243	279	230	198	209	208	215	260	258	231	0.484
Rate of newborns, *n* (‰)													
CP-EOS	19 (0.55)	4 (1.27)	3 (0.91)	1 (0.30)	3 (0.90)	0 (0.00)	2 (0.61)	2 (0.64)	0 (0.00)	3 (0.92)	0 (0.00)	1 (0.35)	0.156
No sepsis	1,805 (52)	245 (78)	175 (53)	200 (61)	166 (50)	123 (38)	149 (45)	149 (48)	148 (46)	175 (54)	148 (57)	127 (44)	0.243
All-cause deaths, *n*, (‰)													
All newborns	124 (3.58)	10 (3.16)	10 (3.05)	11 (3.34)	8 (2.41)	6 (1.86)	13 (3.94)	10 (3.18)	13 (4.04)	16 (4.90)	13 (5.00)	14 (4.87)	0.032
Deaths among treated, *n*, (%)													
All treated	31 (1.70)	1 (0.40)	1 (0.56)	4 (1.99)	3 (1.78)	3 (2.44)	3 (1.97)	6 (3.95)	1 (0.68)	3 (1.68)	2 (1.31)	4 (3.13)	0.372
EOS	1 (5.30)	0 (0)	0 (0)	0 (0)	0 (0)	0 (0)	0 (0)	1 (50)	0 (0)	0 (0)	0 (0)	0 (0)	0.752
No sepsis	30 (1.66)	1 (0.40)	1 (0.57)	4 (2.00)	3 (1.81)	3 (2.44)	3 (2)	5 (3.33)	1 (0.68)	3 (1.70)	2 (1.31)	4 (3.15)	0.372

CP-EOS, culture-proven early-onset sepsis; NA, not applicable.

CP-EOS was defined as sepsis within 6 days of birth (day 0–6).

Categorical and continuous variables are displayed as frequencies (percentages), and as median (IQR), respectively.

a *p*-value calculated with trend test.

The number of deaths among all livebirths was 124, corresponding to an all-cause mortality rate of 3.58 per 1,000 livebirths (95% CI, 2.98–4.27 ‰). Among antibiotic-exposed neonates, the all-cause mortality rate was 1.7% (31/1,824 neonates; 95% CI, 1.2%–2.4%). Mortality was 5.3% (1/19 neonates; 95% CI, 0.1–26.0%) among neonates with CP-EOS and 1.7% (30/1,805 neonates; 95% CI, 1.1%–2.4%) among those without proven EOS (Table 2). Altogether, one newborn died of CP-EOS among 34,668 livebirths, corresponding to 0.03‰ (95% CI, 0.001–0.16‰). Among the 31 deaths that occurred during the first postnatal week, the leading cause of mortality was severe congenital malformations, accounting for 19 deaths (61%), followed by perinatal asphyxia (3/31, 10%).

Among the 19 CP-EOS cases, the most common pathogens were Group B Streptococcus (6/19, 32%) followed by *Escherichia coli* (3/19, 16%) (Supplementary Table S1). Notably, methicillin-resistant *Staphylococcus aureus* (MRSA) was identified in 2 cases (11%), representing the third most frequent pathogen in our cohort. The only infant with CP-EOS who died had intestinal atresia and developed *Escherichia coli* sepsis with septic shock.

## 4 Discussion

In this single-center cohort study including more than 34,000 late-preterm and term newborns over an 11-year period, we found that antibiotic exposure during the first postnatal week remained disproportionally high compared to the burden of CP-EOS. Encouragingly, both antibiotic utilization and the incidence of CP-EOS declined markedly over time, suggesting progress toward more targeted antibiotic use in this population. Overall, 5.3% of newborns received intravenous antibiotics during the first postnatal week, corresponding to 238 antibiotic days per 1,000 livebirths. For each CP-EOS case, 96 newborns received antibiotics and 433 antibiotic days were administered. These findings provide contemporary Canadian benchmarking data on neonatal antibiotic exposure using standardized stewardship indicators and demonstrate that substantial reductions in antibiotic exposure may be achievable without increases in EOS-associated mortality.

Among newborns ≥34 weeks' gestation, CP-EOS is an infrequent event, although incidence rates vary across high-income countries. Recent studies have reported an incidence of CP-EOS ranging from 0.13 to 1.45 per 1,000 livebirths (3, 6, 8, 10, 11, 28, 30, 31). Consistent with these observations, we found a CP-EOS incidence of 0.55 per 1,000 livebirths. Although the EOS-associated mortality rate in our cohort was 5.3%, this estimate should be interpreted with caution given the small number of CP-EOS cases and deaths. Reported EOS-associated mortality rates among late-preterm and term newborns vary across studies, ranging from 1.39% in a Swedish nationwide population-based cohort including more than one million infants (10) to 3.2% in the international AENEAS study including almost 760,000 liveborn late-preterm and full-term newborns (11). Because EOS-associated mortality has become a rare event in late-preterm and term newborns, very large population-based studies including hundreds of thousands of infants are required to provide precise estimates of mortality risk. Importantly, most deaths during the first postnatal week were attributable to causes other than EOS, with severe congenital malformations accounting for the majority, followed by perinatal asphyxia. This is consistent with previous studies showing that congenital malformations and perinatal asphyxia account for more deaths than sepsis among late-preterm and term neonates (32). Notably, the only infant who died with CP-EOS had an underlying congenital malformation (intestinal atresia) and developed *Escherichia coli* sepsis complicated by septic shock. However, the contribution of CP-EOS and the underlying congenital condition to the fatal outcome could not be determined.

Precise quantification of neonatal antibiotic exposure is essential to guide antimicrobial stewardship efforts and facilitate benchmarking across centers (11, 15, 26). In contrast with previous studies focusing primarily on EOS incidence or antibiotic initiation alone, our study simultaneously evaluated the burden of disease and the burden of treatment using standardized indicators proposed by the AENEAS collaboration (11). This approach allows antibiotic exposure to be interpreted in relation to actual infection burden rather than as an isolated metric.

In the AENEAS study, the overall international median of antibiotic exposure was lower than in our cohort; however, AENEAS included heterogeneous networks with wide differences in antibiotic exposure, ranging from low-exposure population-based systems to higher-exposure hospital-based networks (11). Therefore, our results are not an outlier when compared with the full range of AENEAS networks, but they fall toward the higher-exposure end of the spectrum (Supplementary Figure S1). This is clinically relevant because our CP-EOS incidence was broadly comparable to international estimates, suggesting that differences in antibiotic exposure are likely driven more by local management practices than by substantially higher infection burden.

Comparison with the Swedish SWENAB cohort also requires careful interpretation. Unlike our single-center tertiary cohort, SWENAB provides nationwide population-based data across all levels of neonatal care in Sweden (10). Nevertheless, even after stratification by level of care, Swedish level II and III units reported lower proportions of antibiotic-treated newborns and lower antibiotic exposure than observed in our cohort. Although differences in patient complexity, referral patterns, and case mix likely contribute to this discrepancy, these findings suggest that substantial variability exists in the management of suspected EOS, even among higher-acuity neonatal settings.

Several factors may explain the antibiotic exposure observed in our cohort. The management of suspected neonatal EOS remains particularly challenging because the condition is rare but potentially life-threatening, while early clinical manifestations are frequently subtle and nonspecific. In the absence of sufficiently accurate diagnostic tools to reliably exclude infection immediately after birth, empirical antibiotic therapy is often initiated while awaiting microbiological confirmation. In addition, CHUSJ is a tertiary perinatal center caring for newborns with more complex medical conditions and higher-risk perinatal situations, which may contribute to a lower threshold for evaluation and treatment of suspected EOS. This likely introduces a degree of referral and case-mix bias when comparing our results with broader population-based cohorts.

Importantly, however, we observed substantial reductions in antibiotic exposure over time. Between 2013 and 2023, the proportion of treated newborns decreased by 44%, while antibiotic exposure decreased by 21%. This decline was not linear, with fluctuations in antibiotic use observed across individual years. Interestingly, the lowest proportions of antibiotic-treated newborns and the lowest antibiotic exposure were observed between 2017 and 2020, despite relatively stable annual birth rates. Consistent with our findings, a nationwide population-based study from Norway evaluated whether national and local antimicrobial stewardship initiatives were associated with reductions in neonatal antibiotic use and demonstrated substantial decreases in both antibiotic exposure and treatment duration over time (33). Although our study was not designed to identify factors associated with these temporal changes, this pattern may reflect gradual evolution in local clinical practices and antimicrobial stewardship approaches in the management of newborns with suspected EOS. The impact of perinatal GBS screening prevention strategies over time could also contribute to changes in management of suspected neonatal EOS. Because no formal evaluation of stewardship interventions or guideline modifications was performed, these hypotheses remain speculative.

Our findings have important clinical implications. They suggest that additional reductions in antibiotic exposure may be achievable without compromising safety, particularly by targeting antibiotic initiation in clinically stable newborns at low risk of EOS. Strategies such as structured serial clinical examinations, standardized discontinuation protocols, local audit and feedback, and possibly implementation of EOS risk assessment tools could help further align antibiotic use with true infection burden (14, 28, 34–36). The goal is not to avoid antibiotics in newborns who need them, but to reduce unnecessary exposure among those in whom infection is unlikely.

This study has several strengths, including its large sample size, 11-year duration, use of microbiology and pharmacy data, and application of standardized benchmarking indicators. These features allow comparison with international cohorts and provide a baseline for future Canadian neonatal stewardship initiatives.

A number of limitations should also be acknowledged. First, this was a retrospective single-center study. Second, as a tertiary hospital-based cohort, our population may not be directly comparable to population-based studies. Third, we did not evaluate maternal risk factors, clinical indications for antibiotic initiation, illness severity, or specific changes in local guidelines over time. Therefore, we cannot determine which factors directly caused the observed reduction in antibiotic exposure. Fourth, the small number of CP-EOS cases limits precision for mortality estimates. In addition, underlying conditions such as congenital malformations, perinatal asphyxia, or inborn errors of metabolism may contribute to mortality and complicate the attribution of death to EOS, particularly in individual cases. Moreover, our definition of CP-EOS included infections diagnosed within the first seven postnatal days, whereas some studies define EOS within the first 72 h, which may affect direct comparisons. However, the 7-day definition has been used in several large international cohorts, facilitating benchmarking and comparison of our findings with contemporary multicenter data (10, 11). Finally, our study did not evaluate culture-negative sepsis or associated short-term clinical outcomes, which were beyond the scope of the present analysis. Culture-negative sepsis represents a heterogeneous group of clinical conditions, and its diagnosis may be influenced by factors such as blood culture sampling practices (37). Further evaluation of culture-negative sepsis definitions and associated outcomes is needed to better understand the relationship between antibiotic exposure patterns and the broader spectrum of neonatal infections.

## 5 Conclusions

In conclusion, antibiotic exposure for suspected EOS at our Canadian tertiary center decreased substantially over time but remained high relative to the burden of culture-proven infection. Comparison with international benchmarking data suggests that differences in antibiotic use are not explained by EOS incidence alone and may reflect modifiable local practices. These findings support continued neonatal antimicrobial stewardship efforts focused not only on shortening antibiotic duration, but also on improving risk stratification and reducing unnecessary treatment initiation.

## Data Availability

The raw data supporting the conclusions of this article will be made available by the authors, without undue reservation.
