# Supplementary material for: Antibiotic exposure for suspected early-onset sepsis in late-preterm and full-term newborns in Quebec: a 11-year single-center cohort study
Source: Front Pediatr. 2026 Sep 9;14:1922279. doi: 10.3389/fped.2026.1922279 (PMC13597844; doi:10.3389/fped.2026.1922279)
Supplement: Supplementary file 1 [file Supplementaryfile1.docx]

Supplementary Material

# 1 Supplementary Tables and Figures

## 1.1 Supplementary Table 1. Distribution of pathogens in culture-proven early-onset sepsis cases

|  | **CP-EOS**  **(n=19)** |
| --- | --- |
| **Main pathogens, n (%)**  **Gram positive**  Group B Streptococcus  Staphylococcus aureus (MRSA)  Enterococcus faecalis  Streptococcus pneumoniae  Other  **Gram negative**  Escherichia coli  Citrobacter sedlakii  Morganella morganii | **14 (74)**  6 (32)  2 (11)  2 (11)  1 (5)  3 (16)  **5 (26)**  3 (16)  1 (5)  1 (5) |

**1.2 Supplementary Figure 1:** Benchmarking of antibiotic exposure, early-onset sepsis incidence, and birth volume across neonatal networks participating in the Antibiotic Exposure for Suspected Neonatal Early-onset Sepsis (AENEAS) study and the Sainte-Justine cohort.

Each bubble represents a neonatal network in the AENEAS study. The x-axis shows the incidence of culture-proven early-onset sepsis (EOS) per 1,000 live births, and the y-axis shows antibiotic exposure expressed as antibiotic days per 1,000 live births. Bubble size is proportional to the number of live births included in each network. Purple bubbles represent networks participating in the AENEAS study, while the red bubble represents the Sainte-Justine cohort. Dashed lines indicate the overall AENEAS study averages for EOS incidence and antibiotic exposure.
